# Latent and Abnormal Functional Connectivity Circuits in Autism Spectrum Disorder

**DOI:** 10.3389/fnins.2017.00125

**Published:** 2017-03-21

**Authors:** Shuo Chen, Yishi Xing, Jian Kang

**Affiliations:** ^1^Department of Epidemiology and Biostatistics, University of MarylandCollege Park, MD USA; ^2^Department of Biostatistics, University of MichiganAnn Arbor, MI, USA

**Keywords:** Autism spectrum disorder, biomarker, brain connectivity, fMRI, graph topology, network

## Abstract

Autism spectrum disorder (ASD) is associated with disrupted brain networks. Neuroimaging techniques provide noninvasive methods of investigating abnormal connectivity patterns in ASD. In the present study, we compare functional connectivity networks in people with ASD with those in typical controls, using neuroimaging data from the Autism Brain Imaging Data Exchange (ABIDE) project. Specifically, we focus on the characteristics of intrinsic functional connectivity based on data collected by resting-state functional magnetic resonance imaging (rs-fMRI). Our aim was to identify disrupted brain connectivity patterns across all networks, instead of in individual edges, by using advanced statistical methods. Unlike many brain connectome studies, in which networks are prespecified before the edge connectivity in each network is compared between clinical groups, we detected the latent differentially expressed networks automatically. Our network-level analysis identified abnormal connectome networks that (i) included a high proportion of edges that were differentially expressed between people with ASD and typical controls; and (ii) showed highly-organized graph topology. These findings provide new insight into the study of the underlying neuropsychiatric mechanism of ASD.

## 1. Introduction

Autism spectrum disorder (ASD) is a neurodevelopmental disorder whose clinical symptoms include impaired social communication and language abilities, and repetitive behaviors (American Psychiatric Association, 2013). Its prevalence is increasing; one in 68 children were diagnosed with ASD in the United States in 2014 (CDC reports, 2014). However, the etiology of ASD remains unclear. Many recent studies have focused on the neural pathophysiology of brain structures and functions associated with ASD symptoms.

Neuroimaging techniques provide noninvasive methods of studying the neuropathology of ASD by learning about abnormal connectivity patterns. Mounting evidence suggests that ASD is associated with disturbances of neural connectivity rather than solely local neural activities (Di Martino et al., 2014; Hahamy et al., 2015). Resting-state functional magnetic resonance imaging (rs-fMRI) has become widely used to measure the functional connectivity between brain regions by calculating the correlations between time series of spontaneous low-frequency fluctuations in cerebral blood flow. The Autism Brain Imaging Data Exchange (ABIDE) consortium has contributed a publicly available set of existing rs-fMRI data from more than 1000 subjects, with the aim of improving the quality and reliability of functional connectivity research in ASD (Di Martino et al., 2014; Cheng et al., 2015). Many studies have yielded interesting, yet controversial, findings of altered connectivity patterns (Shih et al., 2010; Vissers et al., 2012; Chen et al., 2015a; Ecker et al., 2015; Ha et al., 2015; Hahamy et al., 2015). For example, hypoconnectivity is associated with ASD, particularly in long-range and cross-hemispheric connections, such as those between the left and right insula and left and right parieto-occipital regions, which are known as the default mode network (DMN) (Broyd et al., 2009; Anderson et al., 2010; Schipul et al., 2011; Just et al., 2012; Di Martino et al., 2014). However, these claims have been challenged by findings reporting hyperconnectivity within networks (including the DMN, and frontostriatal, frontotemporal, motor, visual, and salience networks), as well as between the striatum, insula, and superior temporal gyrus, in children with ASD compared with typical children (Di Martino et al., 2011; Müller et al., 2011; Keown et al., 2013; Lynch et al., 2013; Supekar et al., 2013; Uddin et al., 2013).

The conflicting evidence regarding differentially expressed connectome features may arise for many possible reasons, such as demographic variation between subjects recruited in the studies, preprocessing steps, network selection methods, and statistical analysis methods. Recently, Cheng et al. (2015) report reduced connectivity in ASD based on the ABIDE data (418 autism and 509 matched healthy controls) using a voxel-wise meta-analysis, and more importantly they also report that the reduced connectivity is significantly correlated with symptom severity. Building on these findings, we aim to further investigate whether the disrupted brain connections in ASD are systematically organized from a network perspective. However, the disrupted networks in ASD are not known prior to the experiment, making it even more challenging to examine them with statistical rigor.

Conventionally, seed voxel analysis, descriptive statistics and mass univariate analysis are used for group-level brain connectivity analyses (Yeo et al., 2011; Craddock et al., 2013; Sporns, 2014; Smith et al., 2015). Descriptive graph metrics denote brain regions as nodes, and connections between them as edges, and have yielded many interesting findings (Bullmore and Sporns, 2009; Rubinov and Sporns, 2010; Biswal et al., 2010; Achard et al., 2012; Crossley et al., 2013, 2014; Fornito et al., 2013, 2015; van den Heuvel and Sporns, 2013; Stam, 2014). However, such metrics (i.e., modularity, clustering coefficients, and rich-club coefficients) summarize all edges as individual measures and lose localized connectivity (edge-specific) information. Thus, they may lack specificity and sensitivity, making it difficult to interpret such data clinically (Simpson et al., 2015). Mass univariate analysis (e.g., network-based statistics—NBS and family-wise error control; Zalesky et al., 2010), based on the connectome of the whole brain or prespecified brain regions, retains localized information about differentially expressed features but is subject to the trade-off between false positives and a lack of statistical power, and does not account for organized or complex network properties.

Our goal is to detect the latent and abnormal networks that (i) exhibit well-organized topology; and (ii) have a high proportion of differentially expressed edges (hypo- and/or hyperconnections). This approach integrates topological, differentially expressed, and localized edge features, to identify altered connectivity patterns. Recently, network object-oriented algorithms have been developed to detect and test these hidden disease-related brain connectivity networks (Chen et al., 2015b, 2016).

Here, we apply these recently developed statistical techniques to the ABIDE rs-fMRI data sets. Using these new statistical graph methods, our aim was to ASD related abnormal connectivity networks by automatically detecting latent networks with well-organized topological structures. Our resulting edgewise findings converge with previous studies using ABIDE data sets (Cheng et al., 2015). Moreover, we detect networks showing idiosyncratic distortion (Hahamy et al., 2015), which may help uncover the underlying mechanisms responsible for the joint hypo- *and* hyperconnectivity observed in ASD in many topological organization studies. Our findings may improve the understanding of neuropathological machinery and identify biomarkers that assist with disease diagnosis and treatment selection.

## 2. Materials and methods

### 2.1. Data sets and preprocessing

The data set was collected at the University of Michigan, one of the ABIDE data collection sites (Monk et al., 2009; Weng et al., 2010; Di Martino et al., 2014). The publicly available data set comprises data from 48 people with ASD and 65 TCs, with no significant differences in demographics between the two groups. For example, the mean age of the people with ASD at scan was 13.85 years (standard deviation (sd) = 2.31; range, 9.2–18.6); the mean age of the TCs was 15.03 (sd = 3.66; range, 8.2–28.8). Thirty-nine of the 48 people in the ASD group were male, compared with 49 of the 65 TCs. The *p* values from the Wilcoxon rank sum test (age) and Pearson χ^2^ test (sex) were both greater than the α level at 0.05. The study was approved by the local institutional review boards, and data were fully de-identified by removing all 18 Health Insurance Portability and Accountability (HIPAA)-protected health information identifiers as well as facial information from structural images, and data were carefully examined before release to the public (Di Martino et al., 2014). TCs had no behavioral or mental concerns; inclusion and exclusion criteria for TCs are described on the ABIDE project website (http://fcon_1000.projects.nitrc.org/indi/abide/), Typical controls (TCs) were included by the criteria that either verbal or non-verbal IQ was ≥ 85 and were aged at least 7 years, whereas TCs were excluded for those who received a score of 10 or higher on the Social Communication Questionnaire 14 or a score of 6 or higher on the Obsessive/Compulsive subscale of the Spence Children's Anxiety Scale (SCAS) 16.

Imaging was performed on a 3 Tesla GE Signa scanner. Data were obtained using a gradient echo T2^*^-weighted echo planar imaging sequence, echo time = 30 ms, repetition time = 2,000 ms, 64 × 64 matrix with 40 slices, each 4.0 mm thick, no skip, resulting in whole brain coverage with a voxel size of 3.4 × 3.4 × 3.0 mm. During the scan, all subjects were asked to lie as still as possible, keep their eyes open, look at a fixation cross, and to try not to think about anything in particular.

On these rs-fMRI data we performed preprocessing based on the Configurable Pipeline for the Analysis of Connectomes (C-PAC, http://fcp-indi.github.io). The images were slice-time and motion corrected. The data were then registered to a standard Montreal Neurological Institute (MNI) space with voxel size 2 mm^3^ and converted to percent signal change. Masks of white matter, gray matter and cerebrospinal fluid (CSF) were created in the standard MNI space. The mean time series of the white matter, CSF and the six movement parameters were regressed from the gray matter. The linear trend was removed from the signal, and the fMRI time series were bandpass filtered (0.009–0.08 Hz) and spatially smoothed with a 6 mm full width at half maximum Gaussian kernel. Using automated anatomical labeling (AAL), we then used the first 90 regions of interest (ROIs) as nodes (Tzourio-Mazoyer et al., 2002), and took the weighted average of the temporal profiles of all voxels within each ROI as the region level signal for all subjects. The Pearson correlation coefficients were calculated between the 90 nodes and then Fisher's *Z* transformation was performed on each correlation. In our analysis, we focused on detecting and testing alterations in connectivity networks by comparing connectivity matrices between TCs and people with ASD.

### 2.2. Group level analysis

The goal of group-level functional connectivity analysis is to examine whether different groups (or individuals) show differences in connectivity. Conventional brain connectivity and network methods are conducted from two distinct perspectives: testing which edges are differentially expressed, or whether the global graph descriptive metrics differ (Simpson and Laurienti, 2016). Hybrid analyses are more attractive because they enable the identification of well-organized (systematic) networks (subgraphs) where most contained edges are differentially expressed. Such findings may provide insight into systematic disruptions of the brain connectome in people with ASD. To achieve this goal, we used network object-oriented algorithms (Chen et al., 2015b, 2016).

We first compared the TC and ASD data by performing two-sample *t* tests on each of the 4005 edges. Whole-brain results were denoted as a graph, *G* = (*V, E*), where the node set *V* represents a brain region, and an edge *e*_*ij*_ ∈ *E* connects regions *i* and *j*. For each edge (*e*_*ij*_) we assigned the weight as *W*_*ij*_ = −log(*p*_*ij*_). The greater the *W*_*ij*_ value, the greater the difference in this edge between the TC and ASD data. Thus, the weighted adjacency matrix **W** is our input data for the detection of altered networks.

Next, we applied parsimonious differential brain connectivity network detection (Pard, for community detection) and *k*-partite algorithms (Chen et al., 2015b, 2016). The joint use of these algorithms enabled the automatic detection of latent abnormal networks with organized clique and *k*-partite graph topology. For each altered network detected, we performed a permutation test to obtain the statistical significance (network-level *p*-value).

We specified null and alternative hypotheses for testing differentially expressed connectivity networks (Chen et al., 2016). *H*_0_: There is no altered connectivity network when comparing the connectivity matrices across clinical subpopulations; this is equivalent to: (i) there are no differentially expressed edges (C1), or (ii) there *are* differentially expressed edges but they are randomly distributed in the graph *G* (C2). *H*_1_: There are altered connectivity networks; this is equivalent to: (i) there are differentially expressed edges, or (ii) the differentially expressed edges are *not* distributed randomly in the graph *G*, but in an organized pattern.

Therefore, the statistical significance of an altered connectivity network is determined by two factors: (1) the significance levels of all individual edges within the network; (2) the distribution of the differentially expressed edges in *G*. If C2 in the null hypothesis is true and differentially expressed edges are distributed randomly in *G*, then the detected network/subgraph *G*_*k*_ ⊂ *G* is expected to contain a similar proportion of differentially expressed edges in *G*. Thus, based on the combinatorics and graph theory, the probability that the detected subgraph includes a much larger proportion of differentially expressed edges is extremely low, so we reject the null hypothesis. In theory, there are numerous possible subgraphs with various topological structures in *G* and thus testing detected networks is subject to multiplicity. We accounted for this multiple testing issue by using permutation testing techniques (Nichols and Holmes, 2002). In each permutation, we recorded the detected network with the maximum test statistic, and then calculated the percentiles of observed networks among the maximum test statistics from all permutations. We collected the suprathreshold networks as our resulting object-oriented altered connectivity networks. We set the α level of the permutation test as 0.05.

## 3. Results

We applied the above network analysis procedure to the ABIDE data sets. Below is a summary of the latent differentially expressed networks we identified.

**Figure 1:**
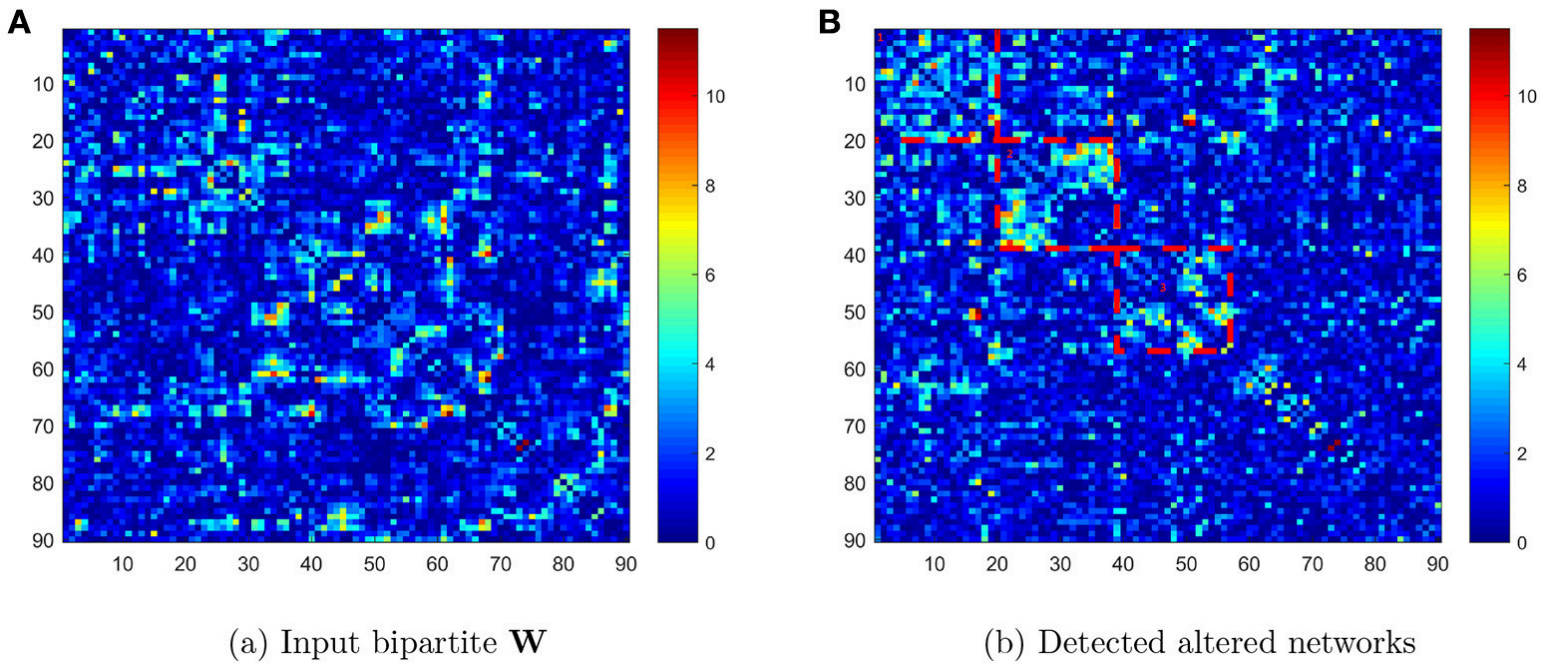
**(A)** Heatmap of −log(*p*_*ij*_) values in the original order of the first 90 AAL regions; **(B)** heatmap of −log(*p*_*ij*_) values reordered to list the detected networks first.

We compared the connectivity metrics (i.e., Fisher's *Z*-transformed correlation coefficients) on each edge between TC and ASD data using two-sample *t* tests, and stored the *p* value as *W*_*ij*_ = −log(*p*_*ij*_) where *i* and *j* were the first 90 AAL brain region indices (*i* ≠ *j* ∈ {1, ⋯, 90}). Figure 1A displays the input data: a 90 × 90 pairwise connectivity testing result matrix (**W**) with the entry *W*_*ij*_ = −log(*p*_*ij*_). The ROIs in the heatmap of Figure 1B are listed in ascending order of regions in the AAL atlas. Next, we applied the Pard algorithm to determine whether the informative edges were distributed in communities, to capture the most differentially expressed edges in parsimonious (clique) networks. We then implemented the *k*-partite graph detection algorithm to obtain multi-partite subgraphs. In the heatmap of Figure 1B, we list the ROIs in order of identified networks and highlight three diagonal blocks, each representing one network. We then performed the permutation test on these networks, which revealed that the first two were significantly different (both *p* < 0.001) whereas the third was not (*p* = 0.068). Therefore, the differentially expressed edges were not randomly distributed in the 90 × 90 graph, but instead they were clustered within well-organized subgraphs.

**Figure 2:**
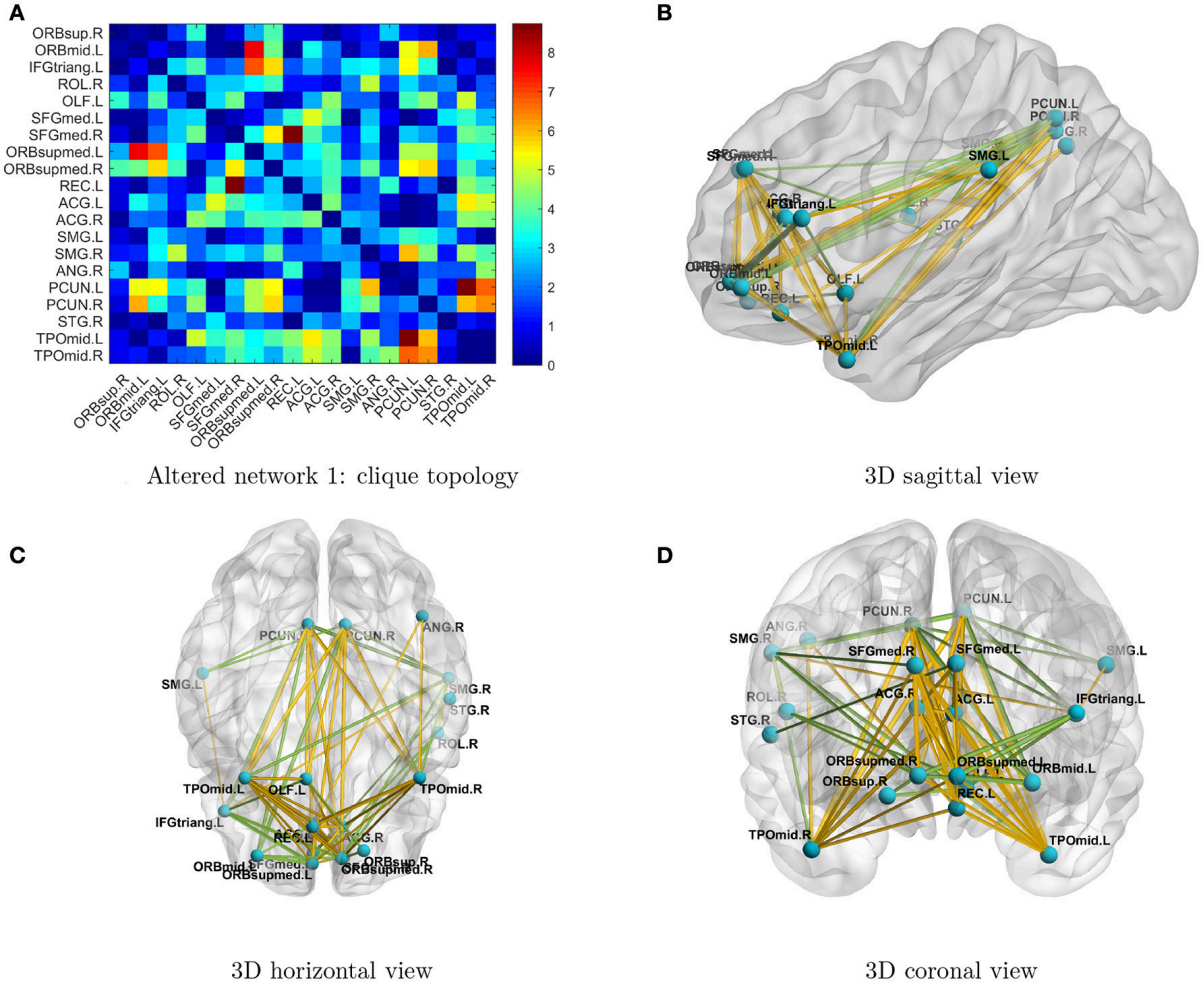
**(A)** Enlarged heatmap showing altered connections between brain regions. **(B–D)** 3D images showing altered connections within the identified network. Yellow edge, TC > ASD; green edge, ASD > TC. The width of an edge reflects the statistical significance of the difference between TC and ASD data.

Next, we investigated the two significant networks in detail. Figure 2 shows the altered connections in the first network in an enlarged heatmap and as 3D images. The region names and corresponding information are listed in Tables 1, 2. The heatmap (Figure 2A) shows the symmetric brain regions related to the altered connections; these include the left and right precuneus (involved in self-consciousness; Margulies et al., 2009), middle temporal gyri (face recognition; Acheson and Hagoort, 2013), supramarginal gyri (empathy; Silani, 2013), superior frontal gyri (self-awareness; Goldberg et al., 2006), and anterior cingulate cortices (emotion Decety and Jackson, 2004). Therefore, the systematic differences may provide a more comprehensive image for us to compare connectomes between TCs and people with ASD. Generally, there are more over-connections across hemispheres in TCs than in people with ASD. Patients with ASD have hypoconnections for most edges linked with right and left middle temporal gyri and precuneus, consistent with that reported by Cherkassky et al. (2006), Anderson et al. (2010), and Lynch et al. (2013). Edgewise comparisons are shown in Supplementary Table 1.

**Table 1:** **Altered network 1 with clique topology**.

**AAL region name**	**Abbreviation**	**Index**	***x***	***y***	***z***
Superior frontal gyrus, orbital part Right	ORBsup.R	6	18	48	−14
Middle frontal gyrus, orbital part, Left	ORBmid.L	9	−31	50	−10
Inferior frontal gyrus, triangular part,Left	IFGtriang.L	13	−46	30	14
Rolandic operculum,Right	ROL.R	18	53	−6	15
Olfactory cortex, Left	OLF.L	21	−8	15	−11
Superior frontal gyrus, medial, Left	SFGmed.L	23	−5	49	31
Superior frontal gyrus, medial, Right	SFGmed.R	24	9	51	30
Superior frontal gyrus, medial orbital, Left	ORBsupmed.L	25	−5	54	−7
Superior frontal gyrus, medial orbital, Right	ORBsupmed.R	26	8	52	−7
Gyrus rectus, Left	REC.L	27	−5	37	−18
Anterior cingulate and paracingulate gyri, Left	ACG.L	31	−4	35	14
Anterior cingulate and paracingulate gyri, Right	ACG.R	32	8	37	16
Supramarginal gyrus, Left	SMG.L	63	−56	−34	30
Supramarginal gyrus, Right	SMG.R	64	58	−32	34
Angular gyrus, Right	ANG.R	66	46	−60	39
Precuneus, Left	PCUN.L	67	−7	−56	48
Precuneus, Right	PCUN.R	68	10	−56	44
Superior temporal gyrus, Right	STG.R	82	58	−22	7
Temporal pole: middle temporal gyrus, Left	TPOmid.L	87	−36	15	−34
Temporal pole: middle temporal gyrus, Right	TPOmid.R	88	44	15	−32

**Table 2:** **Altered network 2 with bipartite topology**.

**AAL region name**	**Abbreviation**	**Index**	***x***	***y***	***z***	**set**
Precentral gyrus, Left	PreCG.L	1	−39	−6	51	2
Olfactory cortex, Right	OLF.R	22	10	16	−11	2
Median cingulate and paracingulate gyri, Left	DCG.L	33	−5	−15	42	1
Median cingulate and paracingulate gyri, Right	DCG.R	34	8	−9	40	1
Posterior cingulate gyrus, Left	PCG.L	35	−5	−43	25	1
Posterior cingulate gyrus, Right	PCG.R	36	7	−42	22	1
Superior occipital gyrus, Left	SOG.L	49	−17	−84	28	2
Superior occipital gyrus, Right	SOG.R	50	24	−81	31	2
Middle occipital gyrus, Left	MOG.L	51	−32	−81	16	2
Middle occipital gyrus, Right	MOG.R	52	37	−80	19	2
Inferior occipital gyrus, Left	IOG.L	53	−36	−78	−8	1
Inferior occipital gyrus, Left	IOG.R	54	38	−82	−8	1
Postcentral gyrus, Right	PoCG.R	58	41	−25	53	2
Superior parietal gyrus, Left	SPG.L	59	−23	−60	59	2
Superior parietal gyrus, Right	SPG.R	60	26	−59	62	2
Inferior parietal, but supramarginal and angular gyri, Left	IPL.L	61	−43	−46	47	2
Angular gyrus, Left	ANG.L	65	−44	−61	36	1
Paracentral lobule, Right	PCL.R	70	7	−32	68	2
Inferior temporal gyrus, Right	ITG.R	90	54	−31	−22	1

We further validate the detected subnetwork features by performing classification analysis. We employ the support vector machine with radial basis function kernel and linear kernel as our classifier. The leave-one-out cross-validation results show that the accuracy rates are 89 and 84% correspondingly.

**Figure 3:**
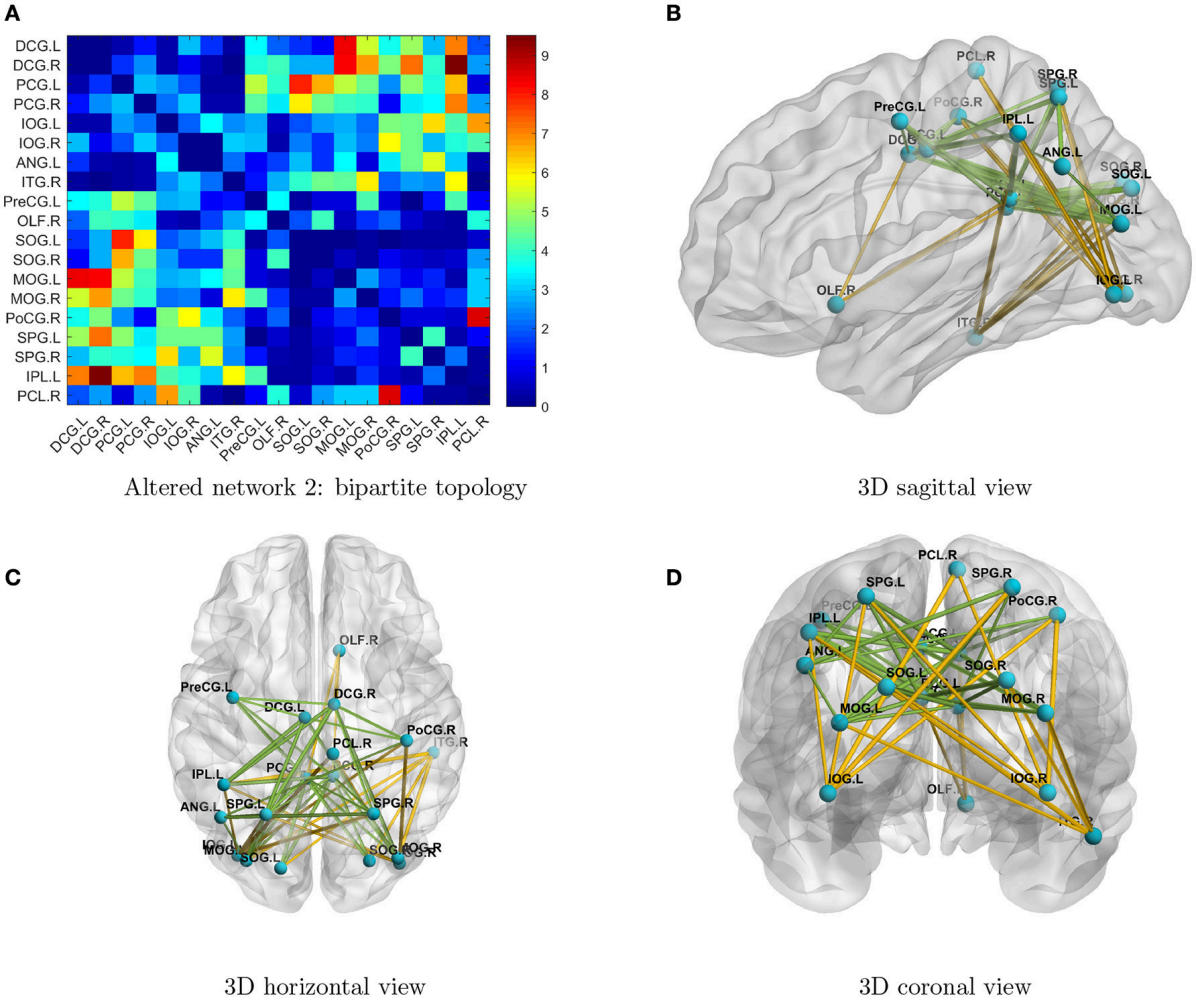
**(A)** Enlarged heatmap showing altered connections between brain regions. **(B–D)** 3D images showing altered connections within the detected network. Yellow edge, TC > ASD; green edge: ASD > TC. The width of an edge reflects the statistical significance of the difference between the TC and ASD groups. Blue nodes, first disjoint set; red nodes, second disjoint set.

By implementing network detection and testing algorithms, we were able to conclude that the differentially expressed edges in the second abnormal connectivity network exhibited a *k*-partite topological structure (the algorithm selected *k* = 2 for this data set). The abnormal connectivity network is shown with a bipartite graph topological structure in Figure 3. In a bipartite graph there two disjoint sets of nodes; edges within each set are less differentially expressed than those between the two sets (Figure 3A). The first set of nodes includes lingual gyri, cingulate gyri, and the left angular gyrus, whereas the second set contains regions from the occipital, parietal, and frontal lobes. Interestingly, the brain regions in the second network are also fairly symmetric. The results suggest that people with ASD have hyperconnections for edges associated with the posterior cingulate gyrus (left and right), and hypoconnections for edges associated with the inferior occipital gyrus (left and right). In addition, all hyperconnections in our ASD group were associated with the angular and cingulate gyrus nodes. The hypo- and hyperconnected edges are in a well-organized topological structure and these results seem to be consistent with those of Monk et al. (2009), Just et al. (2012), Supekar et al. (2013), Uddin et al. (2013), Keown et al. (2013), and Di Martino et al. (2014). Overall, the findings may suggest that the coordination between the visual network (set two) and part of the DMN (set one) may be disrupted. A detailed edgewise comparison table and 3D video are presented as Supplementary Material.

## 4. Discussion

Evidence of abnormal functional connectivity patterns in people with ASD has, to date, been inconsistent. The aim of the present study was to provide a novel strategy for brain connectivity analysis: to simultaneously uncover altered connectivity metrics and network structures. The findings of the present study suggest that unknown, systematic, and organized brain connectivity networks are disrupted in people with ASD. Within these aberrant networks, most edges are differentially expressed between TCs and people with ASD, and these differentially expressed edges show highly organized graph topology. Therefore, in the present study, we consider the altered connectivity as a network unit, rather than as individual edges or global graph summary metrics. This approach has several advantages: (i) the new statistical methods reveal specific nodes and edges within the abnormal networks; (ii) the statistical power is greatly increased while carefully controlling for multiple testing; and (iii) the topology of hypo- and hyperconnections in the abnormal networks could provide insight into the complex machinery underlying ASD. We used advanced graphical statistical methods to detect these hidden disease-related connectivity networks, and performed statistical tests to provide formal inferences.

From a statistical point of view, brain connectivity matrices are intercorrelated, high-throughput data. However, established statistical methods, including multiple-testing adjustment techniques (such as family error and false-positive discovery rate control) and shrinkage techniques (such as least absolute shrinkage and selection operator (Lasso) and elastic net) may not be directly applicable to connectivity analysis. The main reason is that connectivity edges are subject to spatial constraints and are thus dependent on each other in a highly complex, organized, yet unknown, topological structure. Without appropriately accounting for such a dependency structure, we risk a loss of statistical power and possible masking of significant findings. These new network-level connectivity analysis methods (Chen et al., 2015b, 2016) avoid the long-term trade-off between false positive findings and statistical power that arises from the universal cut-off in conventional statistical methods, because the edges borrow statistical strengths from each other through the topological structure. The latent topology provides additional information for statistical modeling and as a result we gain statistical power without increasing false positive error rates.

The topology of detected networks may reveal important underlying neuropathological mechanisms and provide valuable insight for future biological studies. In the networks we identified, most nodes were symmetric across hemispheres, and edges of hypo- and hyperconnections also seemed to be well organized. If we were to perform individual edge statistical analysis, only a small proportion of differentially expressed edges would pass the multiple testing adjustment threshold and no topological patterns would be detected. Interestingly, the two networks we identified include the functional hub nodes of the DMN, such as the posterior cingulate cortex, medial prefrontal cortex, and angular gyri, and the nodes from the dorsomedial subsystem, such as the temporoparietal junction (e.g., inferior parietal lobule and superior temporal gyrus; STG), lateral temporal cortex (e.g., inferior temporal gyrus), and anterior temporal pole (e.g., left and right middle temporal gyri). The first network also involved bilateral anterior, median, and posterior cingulate gyri and the occipital lobes. Our findings largely overlap with previously reported abnormalities in DMN, visual, and motor networks (Di Martino et al., 2011; Just et al., 2012; Uddin et al., 2013; Lynch et al., 2013; Ha et al., 2015; Cheng et al., 2015). The first network is mainly involved in the functional hubs of the DMN and is related to self-consciousness and emotion. The second network reflects the abnormal pattern of connections between parts of the DMN and the visual network. The first and second networks are jointly involved in many features of ASD including those related to receptive language, social cognition, joint attention, action observation, and empathy/emotion. The networks do not identify any consistent hypo- or hyperconnectivity in ASD; instead, the (significant) aberrant connectivities are organized systematically in topological structures. The organized topology of the altered connectivity networks identified here provides further evidence that these findings are promising clinical biomarker candidates.

Throughout this study, we limited our topological structure detection methods to clique and multipartite subgraphs. We are extending these methods to identify various other organized topological structures. We also focused on cross-sectional imaging data and did not address developmental changes of the brain connectome. Although we applied our methods only to fMRI data here, we may further extend these new network-based connectivity analysis tools to various other types of data including functional connectivity data (e.g., from EEG and fMRI) and structural connectivity data (e.g., from diffusion-weighted imaging) to investigate multimodal altered connectivity networks in ASD. The only requirements of the input data are an undirected graph, and that there is no restriction by the choice of connectivity metrics (such as in functional connectivity analysis correlation coefficients, maximum information coefficient, or spectral coherence). In addition, we only utilize a subset of ABIDE data base, and we plan to compare results from different study sites and perform meta-analysis in future work. We also plan to perform multivariate regression analysis to investigate association between brain connectivity and symptom severity at the network level.

We plan to develop more sophisticated algorithms for the automatic detection of complex latent topological structures that have explicit neurological significance, such as rich-club and hyper-graph topology. These new topological structure detection and statistical testing tools have the potential to become important research techniques for understanding the human connectome and its association with neuropsychiatric disorders.

## 5. Author contributions

SC and JK designed this work. SC, JK, and YX performed the data preprocessing and analysis. SC drafted the manuscript.

### Conflict of interest statement

The authors declare that the research was conducted in the absence of any commercial or financial relationships that could be construed as a potential conflict of interest.
